# Translocator Protein Ligand Etifoxine Attenuates MPTP-Induced Neurotoxicity

**DOI:** 10.3389/fnmol.2022.850904

**Published:** 2022-05-24

**Authors:** Qi Tian, Xiaoxia Yang, Juan Du, Huachen Huang, Wei Liu, Peng Zhao

**Affiliations:** Department of Neurology, Tianjin Neurological Institute, Tianjin Medical University General Hospital, Tianjin, China

**Keywords:** MPTP, Parkinson’s disease, neuroinflammation, TSPO, etifoxine

## Abstract

Parkinson’s disease (PD) is a neurodegenerative disease, but the currently available treatments for this disease are symptomatic treatments. There is evidence that translocator protein (18 kDa) (TSPO) expression is upregulated in some neurodegenerative diseases, and TSPO ligands have obvious neuroprotective effects. However, the neuroprotective effects and other potential effects of the TSPO ligand etifoxine in PD remain unclear. Therefore, the present study was designed to explore the impacts of etifoxine on a mouse model of PD induced by 1-methyl-4-phenyl-1,2,3,6-tetrahydropyridine (MPTP). We found that etifoxine significantly reduced motor function deficits, decreased the loss of tyrosine hydroxylase-positive neurons in the substantia nigra, and attenuated the decrease in striatal dopamine levels in mice that received MPTP. Etifoxine diminished the production of inflammatory mediators and infiltration of leukocytes in the brain after MPTP exposure. *In vitro* studies suggested that microglia contribute to etifoxine’s neuroprotective effect. The results showed that etifoxine can alleviate MPTP-induced neurotoxicity and neuroinflammation, providing a new idea for the treatment of PD.

## Introduction

Parkinson’s disease (PD) is the second most common neurodegenerative disease worldwide after Alzheimer’s disease (AD) (Dickson, 2012), and its prevalence is increasing year by year. By 2040, the number of patients with PD worldwide is expected to exceed 12 million. The disease imposes a substantial burden on patients, their families and caregivers, as well as society as a whole (Dowding et al., 2006). The motor symptoms of PD include bradykinesia, hypokinesia and resting tremor, rigidity and gait imbalance while the non-motor features include olfactory dysfunction, cognitive dysfunction, mental symptoms, sleep dysfunction and autonomic nerve dysfunction (Guzman-Martinez et al., 2019). The major pathological feature of the disease is the loss of dopaminergic neurons in the substantia nigra (Lees et al., 2009), and the disease also involves the activation of microglia and an increase in lymphocyte infiltration. Activated microglia can release proinflammatory factors, such as IL-1β, TNF-α, and IL-6, which might eventually cause dopaminergic cell death and promote disease progression (Hirsch and Hunot, 2009; Guzman-Martinez et al., 2019). Currently available treatments address the symptoms of the disease but cannot halt or retard dopaminergic neuron degeneration (Surguchov, 2021). Therefore, brain inflammation has emerged as a new target for the treatment of PD.

Translocator protein (18 kDa) (TSPO) is a five transmembrane domain protein that is localized primarily in the outer mitochondrial membrane. The major function of TSPO is the transport of cholesterol across the membrane for neurosteroid synthesis (Papadopoulos et al., 2006, 2007; Arbo et al., 2015). TSPO is expressed in many organs, and its expression level is higher in tissues containing steroid-synthesizing cells, such as the adrenal glands, gonads, and brain cells, than in other tissues (Papadopoulos et al., 2006). In previous studies, it was observed that TSPO expression was upregulated during brain injury and neuroinflammation (Venneti et al., 2008; Scarf et al., 2009; Rupprecht et al., 2010), and TSPO is mainly expressed in glial cells, especially microglia, with low expression in neurons, the extent of upregulation was consistent with microglia activation, thus the increase of TSPO expression can be regarded as a sign of microglia activation (Chen and Guilarte, 2008). The anti-inflammatory effects of TSPO ligand were observed in both peripheral and central nervous systems (Lee et al., 2016). For example, over-expressed TSPO can improve LPS-induced cognitive deficits in mice, reverse microglia activation and release of pro-inflammatory cytokines (Wang et al., 2016). Therefore, the upregulation of TSPO expression in neuroinflammatory environments suggests that it may be a target for limiting inflammation and dopaminergic neuron death in PD.

Etifoxine (2-ethylamino-6-chloro-4-methyl-4-phenyl-4H-3,1-benzoxazine hydrochloride) is a high-affinity TSPO ligand. Etifoxine has been approved for anti-anxiety therapy (Girard et al., 2012; Poisbeau et al., 2018), compared to traditional benzodiazepines that directly reapply to GABAA receptors, etifoxine affects GABAA receptors by regulating neurosteroid synthesis, at the same time it has less side effects and addiction (Stein, 2015). Considerable evidence indicates that selective TSPO ligands may be therapeutic options for the treatment of inflammatory conditions, reduced infiltration of immune cells, such as brain injury (Simon-O’Brien et al., 2016; Li H. D. et al., 2017; Li M. et al., 2017), multiple sclerosis (Daugherty et al., 2013). Etifoxine can also be proved to improve peripheral nerve injury and nerve pain, the mechanism involved may be the reduction of activated macrophages and their inflammatory products at the site of the lesion (Girard et al., 2008, 2012). However, no studies exploring the potential impact of etifoxine on dopaminergic neuron loss and inflammation in the context of PD have been performed. This study suggests that etifoxine can prevent dopaminergic neuron loss and help attenuate inflammation and motor symptoms in mice exposed to 1-methyl-4-phenyl-1,2,3,6-tetrahydropyridine (MPTP).

## Materials and Methods

### Animals

In this experiment, a total of 96 male 7–8-week-old C57BL/6J (RRID: IMSR_JAX:000664) mice were obtained from Weitong Lihua Experimental Animal (Vital River Laboratory Animal Technology Co., Ltd., Beijing, China). The average body weight was 20–25 g. All the experiments were approved by the Animal Nursing and Use Committee of the Tianjin Medical University General Hospital (Approval No. IBR2019-KY-194), in accordance with the regulations of the National Health Research Institutes for the use of experimental animals. All animals could freely get food and water, and were maintained in a temperature-controlled environment on a 12/12 light-dark cycle. The dead mice did not participate in the experiment, including the Rotarod tests and immunostaining. All animals were deeply anesthetized with 1–2% isoflurane in 100% oxygen for approximately 1–2 min, maintained through a nose cone to minimize animal suffering before killing. In addition, the animal procedures were performed by experienced scientists to ensure that no extra pain was caused to the mice during the experiments. The sample sizes were determined based on prior experimental experience and method described previously. Power analysis was done to verify whether the sample sizes in other tests were large enough to achieve 80% power using the PASS software (α = 0.05). All the results were reported for a minimum of six samples. Details regarding the grouping pattern and number of animals used in each experiment are shown in Figure 2A. According to the ARRIVE (Animal Research: *in vivo* experiment report) guidelines, all animal experiments are designed, performed and reported, and the animals are randomly assigned to the experimental group.

**FIGURE 1:**
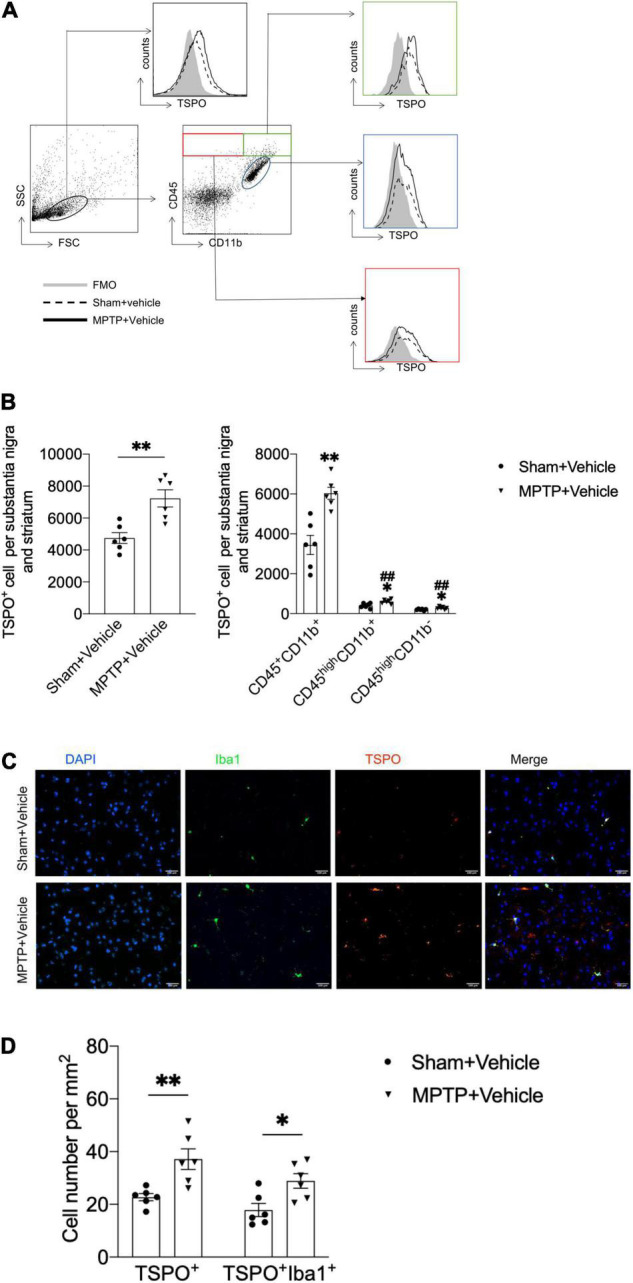
Upregulation of TSPO expression in the brains of MPTP-treated mice. On day 7 after saline or MPTP treatment, single-cell suspensions were prepared from substantia nigra and striatal tissues. **(A)** Flow cytometry plots showing gating of CD45^+^CD11b^+^, CD45^high^CD11b^+^, and CD45^high^CD11b^–^ cell subsets expressing TSPO. **(B)** Bar graph showing the expression of TSPO in the indicated cell subsets. T = 3.916, DF = 10, P = 0.003 (Left). CD45^+^CD11b^+^TSPO^+^: T = 4.572, DF = 10, ^**^P = 0.001; CD45^high^CD11b^+^TSPO^+^: T = 3.058, DF = 10, *P = 0.01, ^##^P < 0.001; CD45^high^CD11b^–^TSPO^+^: T = 2.462, DF = 10, *P = 0.03, ^##^P < 0.001 (Right). *P < 0.05, ^**^P < 0.01 vs. the sham *+* vehicle group for each cell subset. ^#^P < 0.05, ^##^P < 0.01 vs. the MPTP *+* vehicle group for the CD45^+^CD11b^+^ cell subset (n = 6 mice per group). **(C,D)** Immunostaining and summary of the TSPO and Iba1 expression data. Scale bars = 100 mm (n = 3 sections from 6 mice per group). TSPO^+^ cell: T = 3.48, DF = 10, P = 0.006; TSPO^+^Iba1^+^ cell: T = 2.971, DF = 10, P = 0.01. The data are presented as the means ± SEM. *P < 0.05, ^**^P < 0.01 vs. the sham *+* vehicle group for each cell subset.

**FIGURE 2:**
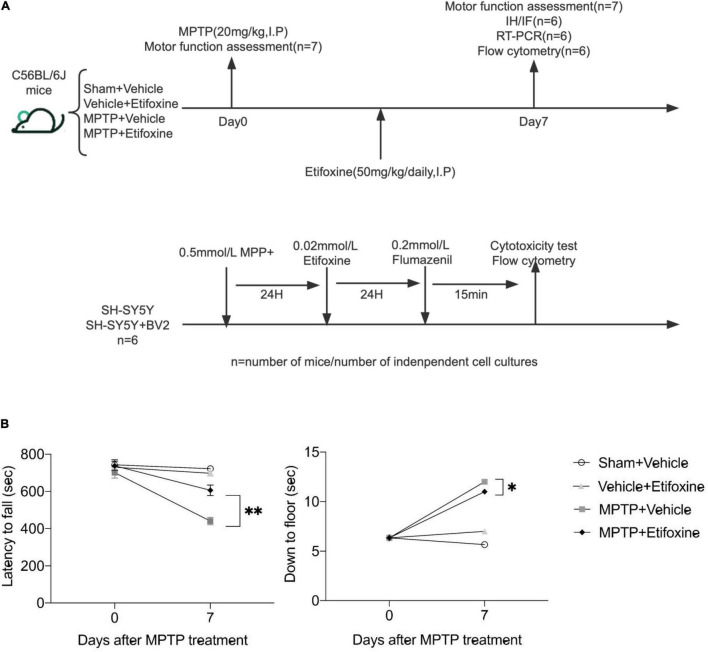
Etifoxine attenuated MPTP-induced motor impairment. **(A)** Schematic showing the experimental design. C57BL/6 mice were administered etifoxine or vehicle daily until the end of the experiment beginning immediately after saline or MPTP administration (intraperitoneal injection). **(B)** Effects of etifoxine on the motor function of C57BL/6 mice following MPTP treatment. *F*(3,48) = 11.86, *P* < 0.001 (Left); *F*(3,48) = 35.98, *P* = 0.024 (Right). Data are presented as means ± SEM. (*n* = 7 mice per group), *P < 0.05, ^**^P < 0.01.

### MPTP-Treatment Mice

C57BL/6J mice received 4 intraperitoneal injections of MPTP-HCl (20 mg/kg, I.P; Cat No. M0896, Sigma-Aldrich, St. Louis, MO, United States) in 2-h intervals. The experiment was performed on the 7 days after the last injection. The mice in the same sex and litter control group were treated with normal saline.

### Drug Administration

The original solution was obtained by dissolving etifoxine [Cat No. HY-16579, MedChemExpress (year 2020)] in DMSO, and the original solution was diluted with normal saline containing PEG-300. After the last injection of MPTP, the mice were immediately injected intraperitoneal with 50 mg/kg etifoxine once a day for 7 days.

### Motor Function Assessments

#### Rotarod Test

The motor ability of the mice was evaluated, especially the ability of coordination and balance. The pre-experiment of turning rods in mice was carried out 2 days before the formal experiment. The mice were placed on an automated accelerating rotarod apparatus (3 cm in diameter and 30 cm long, with a non-slip surface 20 cm above the base), in which the rotation speed of the rotating rod accelerated from 0 to 40 rpm, and the falling time of the mice was recorded for three times in a row, each time at an interval of 30 min. The results are expressed as the average time of the three trials.

#### Pole Test

A pole test was used to determine the degree of bradykinesia. A foam ball with a diameter of 2.5 cm was fixed on the top of a wooden pole (length, 650 cm; width 1 cm), and the head of the mice was placed upward on the top of the ball, and the time required for the mouse to complete the whole climb was recorded. It was carried out for three times in a row, and the result was expressed as the average time of the three experiments.

In this study, the test was carried out on the 0 and 7th day between 9 am and 11 am after etifoxine or vehicle treatment. And treated animals were assessed prior to control animals.

### Immunostaining

After the brain tissue was frozen, it was sectioned into 25-μm-thick slices before fixation with 4% paraformaldehyde for 30 min. To measure the number of tyrosine hydroxylase–positive (TH^+^) cells in substantia nigra, brain sections were incubated with peroxidase sealant for 10 min, followed by 3% BSA for 30 min. Then use primary antibody against TH (RRID: AB_2201528, 1:200, Millipore, Billerica, MA, United States) 4°C overnight, followed by 1 h incubation at room temperature with a biotinylated secondary antibody [Cat No. GK500710; GeneTech, Shanghai, China (year 2020)]. After staining with 3,3’-diaminobenzidine [Cat No. GK500710; 1:50, GeneTech (year 2020)], use neutral balsam to cover sections. A total of three slices were counted in each brain, and each brain slice was separated by 250 μm.

To count the TSPO^+^ cell subsets, brain tissue was frozen and sectioned into 8-μm-thick slices. Brain sections were incubated in blocking solution (5% donkey serum in PBS solution), followed by incubated with goat anti-Iba1 [Cat No. 011-27991, 1:300, Wako, Tokyo, Japan (year 2020)] and anti-PBR (RRID: AB_10862345, Cat No. ab109497, 1:300, Abcam, Cambridge, United Kingdom) 4°C overnight. After triple washings with PBS, the slices were incubated with secondary antibodies: donkey anti-rabbit 594 (RRID: AB_141637, 1:500, Thermo Fisher Scientific, Waltham, MA, United States) and donkey anti-goat 488 (RRID: AB_2534102, 1:500, Thermo Fisher Scientific). After incubation at room temperature for 1 h, the nuclei were stained with DAPI (Abcam). Images were captured by microscope (model BX-61; Olympus Tokyo, Japan) and analyzed using ImageJ software.

### Flow Cytometry

Quantitative analyses of the immune cells prepared from brain tissues and stained with fluorochrome conjugated antibodies followed, as previously described. The mice were killed after 7 days treated with MPTP. The substantia nigra and striatum of mice were isolated and 30 min were digested with papain. The cellular components in brain tissue were separated by 30% percoll, and the cells were stained with fluorochrome-conjugated antibodies. Antibodies were labeled with 1 of 7 fluorescent tags: FITC, BV421, PE, PerCP, APC, APC/Cyanine7, and PE/Cyanine7. The following antibodies were used: CD3 (RRID: AB_2057374, BioLegend, San Diego, CA, United States), CD4 (RRID: AB_312690, BioLegend, United States), CD8 [Cat No. 100728, BioLegend, United States (year 2021)], CD19 (RRID: AB_313654, BioLegend, United States), NK1.1 (RRID: AB_2876525, BioLegend, United States), CD45 (RRID: AB_893343, BioLegend, United States), CD11b (RRID: AB_312798, BioLegend, United States), LY6C (RRID: AB_1732087, BioLegend, United States), Ly6G (RRID: AB_1186104, BioLegend, United States), and PBR (RRID: AB_10862345, Abcam, United States).

SH-SY5Y and BV2 cell lines were treated with drugs and then stained with Annexin-FITC, PI-PE (CA1020, Solarbio) after following the manufacturer’s instructions. Flow cytometric measurements were performed on a FACSAria (BD Biosciences) and analyzed using FACSDiva and FlowJo 9.0 software (FlowJo, Ashland, OR, United States).

### RT-PCR

RNA was extracted from substantia nigra and striatum using TRIzol (Thermo Fisher Scientific). The cDNA was transcribed using StarScript II First-strand cDNA Synthesis Mix (A223-10, GenStar, Beijing, China). PCR is operated on a DNA Engine Opticon 2 real-time PCR detection system (Bio-Rad, Hercules, CA, United States) using 2 × RealStar Green Fast Mixture (A301-10, GenStar, Beijing, China) combined with corresponding primers (Table 1).

**TABLE 1:** Primer sequences for quantitative RT-PCR (Primer, 5′→3′).

Gene	Forward	Reverse
IL-1β	GCTGCTTCCAAACCTTTGAC	AGCTTCTCCACAGCCACAAT
IL-2	ATAACATCCAAGGCATCACCAA	CCACGAGTCTCCTCATAAATCA
IL-4	GCAACGAAGAACACCACAGA	TGCAGCTCCATGAGAACACT
IL-6	ACCGCTATGAAGTTCCTCTCTGCA	AAGCCTCCGACTTGTGAAGTGGT
IL-8	CTGTTGGCCCAATTACTAACAG	TCCCGAATTGGAAAGGGAAATA
TNF-α	ACGGCATGGATCTCAAAGAC	GTGGGTGAGGAGCACGTAGT
IFN-γ	CTTGAAAGACAATCAGGCCATC	CTTGGCAATACTCATGAATGCA
MIP-1α	AGATTCCACGCCAATTCATC	CCCAGGTCTCTTTGGAGTCA
iNOS	ACTACTACCAGATCGAGCCC	GCTAGTGCTTCAGACTTCCC

### Cell Culture and Cytotoxicity Test

SH-SY5Y cells (Cat No. CL-0208) were kindly provided by Procell Life Science & Technology and BV2 (Cat No. 1101MOU-PUMC000063) were obtained from the Chinese Academy of Sciences and maintained in DMEM-F12 medium supplemented with 10% heat inactivated fetal bovine serum, penicillin-streptomycin and L-glutamine. This cell line is not listed as a commonly misidentified cell line by the International Cell Line Authentication Committee. A maximum of four cell passages was used. Cytotoxicity induced by MPP^+^ was measured by using the MTT [3-(4,5-dimethylthiazol-2-yl)-2,5-diphenyltetrazolium bromide] method (m1020, Solarbio, Beijing, China). Absorbance was measured with a microplate photometer (Thermo Fisher Scientific, United States) at a wavelength of 490 nm with a reference wavelength of 630 nm.

### Statistics

Data are expressed as means ± SEM. Statistical analyses were performed with Prism 9.0 software (GraphPad, San Diego, CA, United States). During experiments and analysis, the investigators were blinded to control and experimental group. Animals were identified by earmarks that assigned numbers to each, which were announced to the investigator only after finishing experiments and analysis. Data from the motor function assessments, real-time reverse transcription polymerase chain reaction (RT-PCR), Flow cytometry, immunostaining and cytotoxicity test were analyzed using the Kolmogorov–Smirnov test for normal distribution detection. A two-tailed unpaired Student’s *t*-test was used to determine the significance of differences between two groups. One-way ANOVA followed by Tukey’s *post hoc* test were used for three or more groups. Two-way repeated-measures ANOVA and Bonferroni *post hoc* tests were performed for multiple comparisons. No test for outliers was conducted on the data obtained in the study. A value of *P* < 0.05 indicated significance.

## Results

### Translocator Protein Expression Was Elevated in Parkinson’s Disease Mice

To determine the immune cell types that express TSPO in the context of PD, we prepared single cell suspensions from substantia nigra and striatum tissues obtained from mice with MPTP-induced PD and assessed the expression of TSPO by flow cytometry. Compared with that in the sham + vehicle group, the expression of TSPO in the MPTP + vehicle group was increased. Furthermore, the immune cell type that expressed the highest level of TSPO was CD45^+^CD11b^+^ microglia, which expressed TSPO at a significantly higher level than CD45^high^CD11b^+^ and CD45^high^CD11b^–^ immune cells (Figures 1A,B). In addition, immunofluorescence staining revealed that TSPO was mainly expressed in Iba1^+^ cells (Figures 1C,D). These data showed that the expression of TSPO in the brain tissues of PD mice was increased and that TSPO was mainly expressed in microglia.

### The Translocator Protein Ligand Etifoxine Ameliorates MPTP-Induced Motor Dysfunction

We evaluated the effect of the TSPO ligand etifoxine on the motor function of PD mice. The mice were intraperitoneally injected with etifoxine (50 mg/kg) or vehicle (PEG300, normal saline) for seven consecutive days immediately after injection of MPTP or saline. On days 0 and 7 after etifoxine or vehicle treatment, motor function was assessed by measuring the latency to fall in the rotarod test and the time required to reach the floor in the pole test. Etifoxine alleviated the motor dysfunction caused by MPTP (Figure 2B), showing that etifoxine can ameliorate the motor damage induced by MPTP.

### Etifoxine Significantly Reduces the Loss of Dopaminergic Neurons Induced by MPTP

**FIGURE 3:**
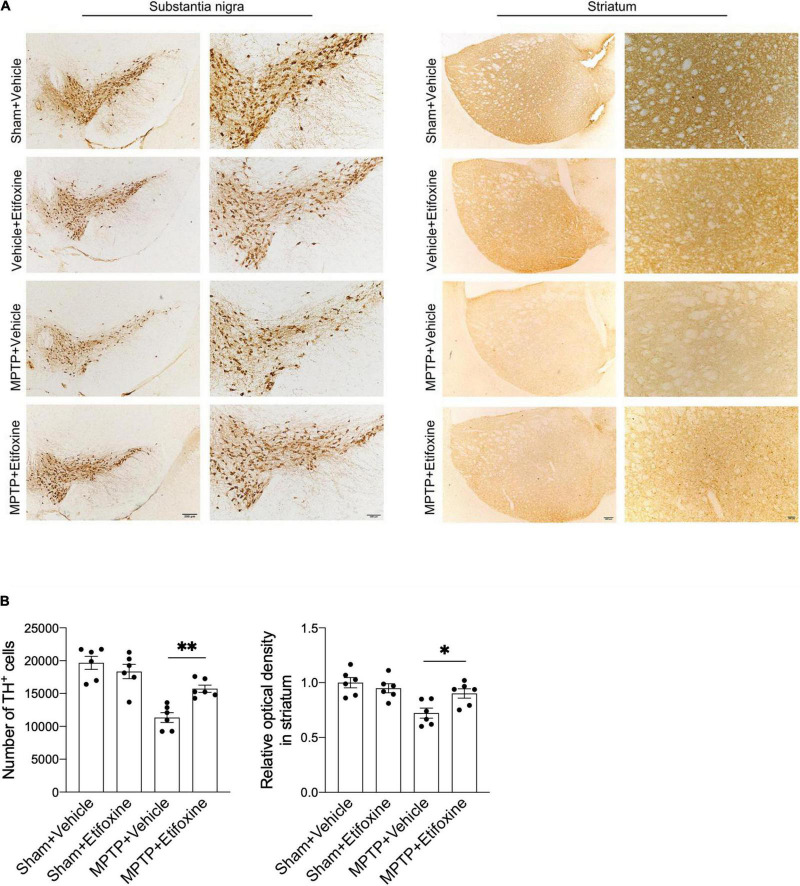
Etifoxine decreased nigrostriatal dopaminergic neuron degeneration following MPTP treatment. **(A)** Immunostaining for TH showing TH^+^ cells in the substantia nigra and TH immunoreactivity in the striata in C57BL/6 mice receiving etifoxine or vehicle on day 7 following saline or MPTP treatment. **(B)** Bar graphs showing the effects of etifoxine on the number of TH^+^ cells in the substantia nigra and TH immunoreactivity in the striatum 7 days following vehicle or etifoxine treatment. *F*(3,20) = 17.88, *P* = 0.009 (Left); *F*(3,20) = 7.452, *P* = 0.04 (Right). All data are presented as means ± SEM. (*n* = 6 mice per group) *P < 0.05, ^**^P < 0.01.

To clarify the effect of etifoxine on MPTP-induced loss of dopaminergic neurons, we assessed the number of TH^+^ cells in the substantia nigra and the optical density of TH immunoreactivity in the striatum in mice treated with either MPTP and etifoxine, saline and etifoxine, vehicle and saline, or vehicle and MPTP (Figure 3A). The number of TH^+^ cells in the substantia nigra in the MPTP + etifoxine group was significantly higher than that in the MPTP + vehicle group. Similarly, the optical density of TH immunoreactivity in the striatum was significantly increased (Figure 3B). This suggests that etifoxine can significantly reduce the loss of dopaminergic neurons induced by MPTP.

### Etifoxine Alleviates MPTP-Induced Leukocyte Infiltration and Proinflammatory Cytokine Production

**FIGURE 4:**
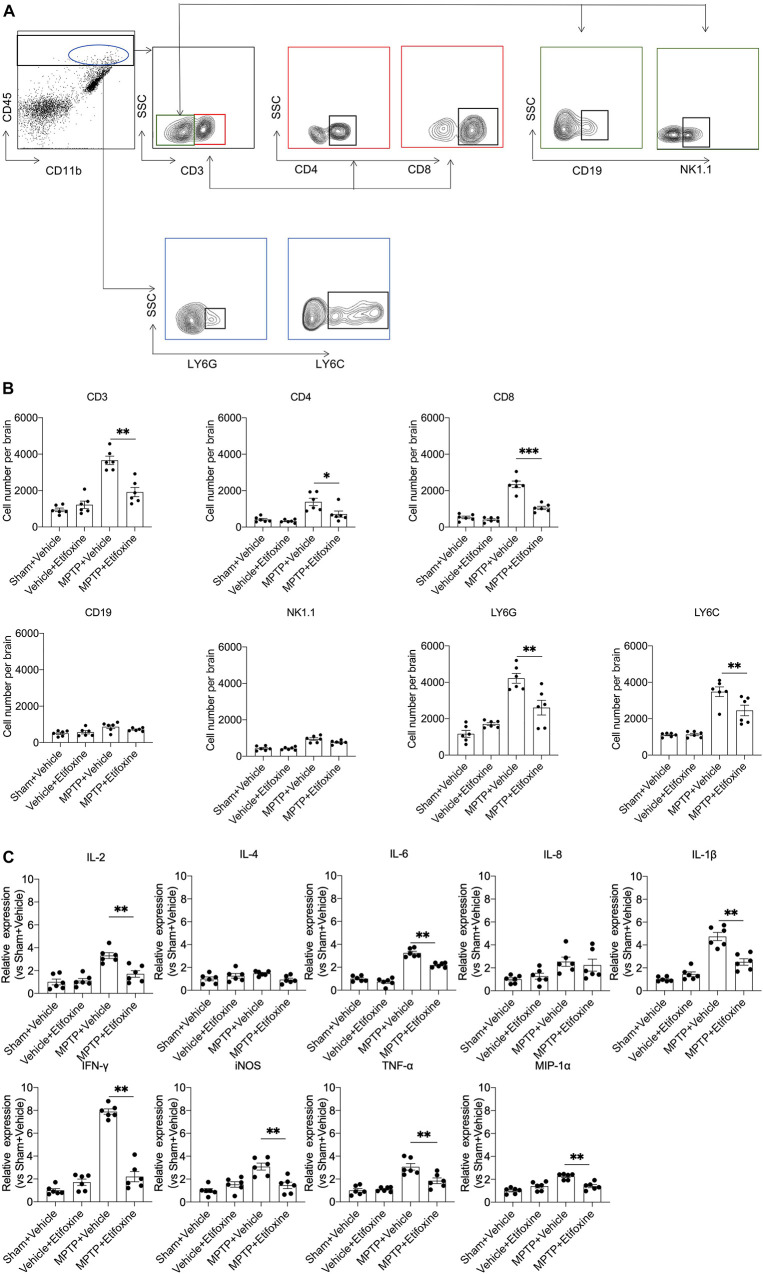
Etifoxine decreased leukocyte infiltration and brain inflammation following MPTP treatment. C57BL/6 mice were administered etifoxine or vehicle immediately after saline or MPTP administration. After day 7, suspensions were prepared from substantia nigra and striatal tissues. **(A)** Flow cytometry plots showing the gating strategy for infiltrating leukocyte subsets, including T cells (CD45^high^CD3^+^), CD4^+^ T cells (CD45^high^CD3^+^CD4^+^), CD8^+^ T cells (CD45^high^CD3^+^CD8^+^), B cells (CD45^high^CD3^–^CD19^+^), NK cells (CD45^high^CD3^–^NK1.1^+^), macrophages (CD45^high^CD11b^+^LY6C^+^), and neutrophils (CD45^high^CD11b^+^Ly6G^+^). **(B)** Quantification of infiltrating lymphocytes, macrophages, and neutrophils in the groups of mice receiving the indicated treatments. T cells: *F*(3,20) = 36.01, *P* < 0.001; CD4^+^ T cells: *F*(3,20) = 12.48, *P* = 0.01; CD8^+^ T cells: *F*(3,20) = 66.51, *P* < 0.001; B cells: *F*(3,20) = 4.483, *P* = 0.51; NK cells: *F*(3,20) = 24.73, *P* = 0.09; macrophages: *F*(3,20) = 32.11, *P* = 0.009; neutrophils: *F*(3,20) = 25.53, *P* = 0.002, *P < 0.05, ^**^P < 0.01. **(C)** Bar graphs showing the mRNA expression of IL-2, IL-4, IL-6, IL-8, IL-1β, TNF-α, MIP-1α, iNOS, and IFN-γ in substantia nigra and striatal tissues from groups of mice receiving the indicated treatment. IL-2: *F*(3,20) = 17.61, *P* = 0.001; IL-4: *F*(3,20) = 2.701, *P* = 0.086; IL-6: *F*(3, 20) = 96.75, *P* < 0.001; IL-8: *F*(3,20) = 4.170, *P* = 0.94; IL-1β: *F*(3,20) = 43.86, *P* < 0.001; TNF-α: *F*(3, 20) = 20.50, *P* = 0.003; MIP-1α: *F*(3,20) = 17.81, *P* < 0.001; iNOS: *F*(3,20) = 12.82, *P* < 0.001; IFN-γ: *F*(3,20) = 111.7, *P* < 0.001. All data are presented as means ± SEM. (*n* = 6 mice per group) *P < 0.05, ^**^P < 0.01.

We wanted to determine the impact of etifoxine on brain inflammation induced by MPTP. Flow cytometry was used to measure the changes in leukocyte infiltration in the substantia nigra and striatum in groups of mice receiving MPTP and etifoxine, etifoxine and saline, vehicle and saline, or vehicle and MPTP. The results showed that etifoxine reduced the infiltration of CD4^+^ T cells (CD45^high^CD3^+^CD4^+^), CD8^+^ T cells (CD45^high^CD3^+^CD8^+^), neutrophils (CD45^high^CD11b^+^LY6G^+^) and macrophages (CD45^high^CD11b^+^LY6C^+^) (Figures 4A,B), which indicated that etifoxine could reduce leukocyte infiltration induced by MPTP. The results of immunofluorescence staining were consistent with those of flow cytometry. In addition, etifoxine treatment decreased the expression of IL-1β, TNF-α, IL-2, IL-6, macrophage inflammatory protein-1α (MIP-1α), IFN-γ, and iNOS (Figure 4C). These results suggest that etifoxine can reduce brain inflammation induced by MPTP.

### Microglia Contribute to the Neuroprotective Effect of Etifoxine

**FIGURE 5:**
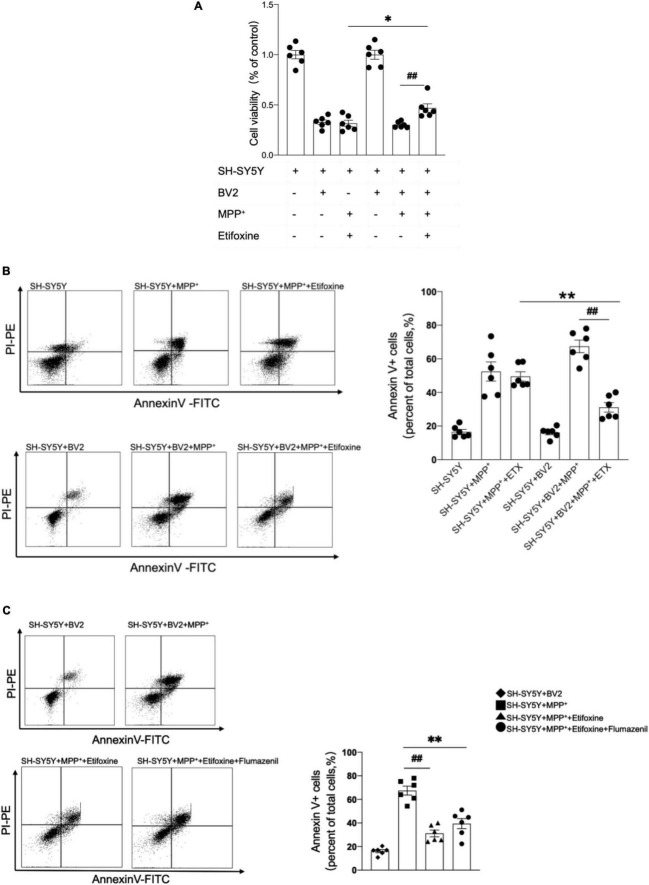
The neuroprotective effect of etifoxine depended on microglia. **(A)** SH-SY5Y cells and BV2 microglia were co-cultured in DMEM-F12 medium and incubated with 0.5 mmol/L MPP^+^ for 24 h then 0.02 mmol/L etifoxine for 24 h. Bar graph showing the activity of SH-SY5Y cells and BV2 cells treated with MPP^+^ and etifoxine. The absorbance was measured at 490 nm, and the reference wavelength was 630 nm. *F*(5,30) = 96.46, ^##^*P* = 0.02, *P = 0.04. **(B)** Flow cytometry plots showing the gating strategy for apoptotic cells (Annexin V^+^ cell). Bar scatter plot to quantify apoptotic cells. *F*(5,30) = 3.345, ^**^P = 0.005, ^##^*P* < 0.001. **(C)** Cells were treated with 0.2 mM flumazenil for 15 min. Scatter plots indicate apoptosis. *F*(3,20) = 2.162, ^**^P < 0.001, ^##^*P* < 0.001. All data were expressed as mean ± SEM. (*n* = 6 of independent cell cultures) *P < 0.05, ^**^P < 0.05.

Microglia are the main cells expressing TSPO in mice with MPTP-induced PD. We further elucidated whether the neuroprotective effect of etifoxine was related to microglia. SH-SY5Y cells were treated with 0.5 mM MPP^+^ for 24 h and with 0.02 mM etifoxine for 24 h. Cell viability was measured with flow cytometry and MTT kit. The results showed that etifoxine could not attenuate damage to SH-SY5Y induced by MPP^+^, while etifoxine could significantly attenuate damage to SH-SY5Y cells co-cultured with BV2 microglia treated with MPP^+^ (Figures 5A,B). The results showed that microglia aided etifoxine in reducing the neurotoxic effects of MPTP on dopaminergic neurons.

To clarify whether flumazenil could antagonize the protective effect of etifoxine, the medium containing 0.2 mM flumazenil was incubated for 15 min after etifoxine treatment, and the flow cytometry results showed that flumazenil could not significantly increase the percentage of Annexin V^+^ cells (Figure 5C), indicating that flumazenil could not antagonize the protective effect of etifoxine.

## Discussion

This study showed that etifoxine alleviates MPTP-induced dopaminergic neuron death and motor dysfunction and inhibits leukocyte infiltration and microglia-mediated secretion of inflammatory factors. In vitro experiments also demonstrated that etifoxine’s neuroprotective effects are dependent on microglia. These findings imply that etifoxine is sufficient to reduce neurotoxicity and alleviate disease prognosis in animal models of MPTP-induced PD. This proposes a novel approach for the treatment of PD.

Currently, there is some evidence that activated microglia play a major role in neuroinflammation in PD (Ransohoff, 2016; Maiti et al., 2017; Emamzadeh and Surguchov, 2018) and may be an early indicator of PD (Choi et al., 2021). Activated microglia can produce a variety of reactive oxygen species (ROS) (Dias et al., 2013). On the other hand, activated microglia release inflammatory cytokines, cause the aggregation of α-synuclein (Gao et al., 2008), the accumulated α-synuclein makes dopamine neurons more sensitive to neuroinflammation, exacerbate dopaminergic neuron death and cause nerve damage in PD while also stimulating microglial activation and promoting a vicious cycle (Block et al., 2007). Furthermore, some research shows that aggregation of α-synuclein in microglia induces dopaminergic neuron degeneration (Bido et al., 2021). The above evidence shows that microglia play an important role in the pathological process of PD (Rupprecht et al., 2010; Giga et al., 2021). Previous studies have shown that microglia are the main source of TSPO in the central nervous system. The results of this study demonstrate that TSPO expression was mainly upregulated in CD45^+^CD11b^+^ microglia in MPTP-induced mice compared with control mice, and the immunofluorescence results also showed that the number of TSPO^+^Iba1^+^ cells was significantly increased, indicating that TSPO could be used as a new target for treating PD.

To further clarify the neuroprotective mechanism of etifoxine against PD, we evaluated brain inflammation. There are several times more microglia in the substantia nigra than in other brain regions (Kim et al., 2000), making the substantia nigra more susceptible to inflammation than other regions. Activated microglia can release proinflammatory cytokines (TNF-α, IL-1β, IL-2, IL-4, and IL-6) and chemokines (MIP-1α and IFN) and further induce the loss of dopaminergic neurons in the substantia nigra. This study is consistent with previous research showing that etifoxine has a neuroprotective effect (Simon-O’Brien et al., 2016; Li H. D. et al., 2017; Li M. et al., 2017; Dimitrova-Shumkovska et al., 2020). These previous studies confirmed that etifoxine can reduce the toxic effects of MPTP on dopaminergic neurons and limit brain inflammation. Reduced release of proinflammatory cytokines and enhancement of neuronal survival have been observed in numerous models of brain damage in previous studies.

Studies have shown that TSPO can control steroid secretion and apoptosis by affecting the production of ROS (Girard et al., 2008). As a source of oxidative stress, nerve cells are more vulnerable to oxidative damage than other cells. The increase in ROS levels in PD can promote the degeneration and death of dopaminergic neurons in the substantia nigra. Etifoxine has been proven to reduce the generation of ROS (Biswas et al., 2018). The results showed that etifoxine can significantly reduce MPTP-induced dopaminergic neuron death in the substantia nigra. Furthermore, etifoxine, as an enhancer of neurosteroid synthesis (Rupprecht et al., 2010), can increase the concentrations of progesterone, pregnenolone, 5α-dihydroprogesterone, and allopregnanolone in the plasma and brain. In the central nervous system, neurosteroids can regulate many neurotransmitter systems, including the inhibitory GABAergic system and excitatory glutamatergic system. Etifoxine directly binds to the GABAA receptor as an active allosteric regulator to enhance GABAergic synaptic transmission (Schlichter et al., 2000). In PD, loss of dopamine caused by the degeneration of neurons in the substantia nigra pars compacta leads to important changes in neurotransmitter circuits in the basal ganglia, and an imbalance between GABA transmission and glutamate transmission leads to an increase in excitation in the substantia nigra pars compacta and promotes excitotoxicity and dopaminergic cell death. Previous experiments have also shown that flumazenil does not antagonize the anxiolytic effect of etifoxine, considering that it may indirectly affect GABAA receptors by regulating neurosteroid synthesis. In the future, we will conduct further studies on the mechanism of neuroprotective effects of etifoxine.

In summary, etifoxine reduces dopaminergic neuron death and ameliorates neurological impairment in an MPTP-induced PD model. These results suggest that etifoxine may be a candidate preclinical drug for the treatment of PD.

## Data Availability Statement

The original contributions presented in the study are included in the article/supplementary material, further inquiries can be directed to the corresponding author/s.

## Ethics Statement

The animal study was reviewed and approved by the Animal Nursing and Use Committee of the Tianjin Medical University General Hospital (Approval No. IBR2019-KY-194).

## Author Contributions

PZ designed the study and wrote and edited the manuscript. QT and XY performed the study. QT, XY, JD, HH, and WL analyzed the data and prepared the manuscript. All authors contributed to the article and approved the submitted version.

## Conflict of Interest

The authors declare that the research was conducted in the absence of any commercial or financial relationships that could be construed as a potential conflict of interest.

## Publisher’s Note

All claims expressed in this article are solely those of the authors and do not necessarily represent those of their affiliated organizations, or those of the publisher, the editors and the reviewers. Any product that may be evaluated in this article, or claim that may be made by its manufacturer, is not guaranteed or endorsed by the publisher.

## Abbreviations

AD: Alzheimer’s disease
BSA: bovine serum albumin
DMSO: dimethyl sulfoxide
FMO: fluorescence minus one
GABA: gamma-aminobutyric acid
IFN: interferon
IL: interleukin
I.P: intraperitoneal injection
MIP: macrophage inflammatory protein
MPTP: 1-methyl-4-phenyl-1,2,3,6-tetrahydropyridine
MPP^+^: 1-methyl-4-phenylpyridinium iodide
MTT: 3-(4,5-dimethylthiazol-2-yl)-2,5-diphenyltetrazolium bromide
PD: Parkinson’s disease
ROS: reactive oxygen species
TH: tyrosine hydroxylase
TNF: tumor necrosis factor
TSPO: translocator protein.
